# Supplementary material for: A Bayesian method to infer copy number clones from single-cell RNA and ATAC sequencing
Source: PLoS Comput Biol. 2023 Nov 2;19(11):e1011557. doi: 10.1371/journal.pcbi.1011557 (PMC10645363; doi:10.1371/journal.pcbi.1011557)
Supplement: S1 Text — (PDF) [file pcbi.1011557.s001.pdf]

# S1 Text

## Inference robustness against priors

By default, we initializes the CNA distribution for each cluster and each segment using the copy number values provided as input. CONGAS+ then updates this vector using a Dirichlet, starting from a prior vector peaked at a ploidy value equal to. The default value is

$$\text{Dirichlet}(0.1, 0.6, 0.1, 0.1, 0.1)$$

to resemble higher probability mass for the diploid state.

We investigated whether changes in the concentration vector of this Dirichlet have an impact on the inference result. We used 10 synthetic datasets with $K = 3$ clusters, and we first ran CONGAS+ using a prior whose weight on the diploid state is equal to 0.6 (default). From this run, we computed the ARI between the inferred cluster labels and the ground truth, and we kept the obtained values as the baseline ARI for each of the 10 replicates. Next, we ran the inference changing the weight given to the diploid state, and we compared the obtained ARI with the baseline (ARI delta). With the exception of a small number of outliers, we observed that the delta is always very close to zero, indicating that changes in the Dirichlet prior do not have a big impact on the inference.

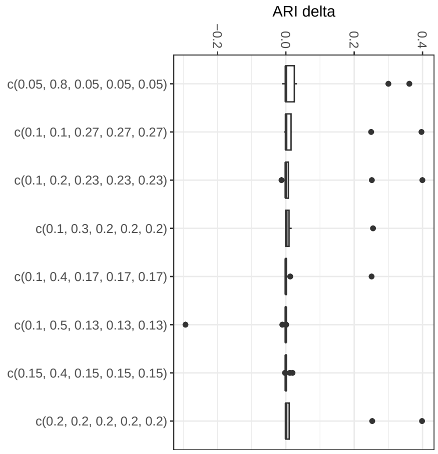


Figure S1: Boxplot showing the difference in ARI obtained on 10 replicates of synthetic tests. The baseline ARI corresponds to CONGAS+ runs with a Dirichlet prior equal to default $(0.1, 0.6, 0.1, 0.1, 0.1)$, and the delta corresponds to the difference between the baseline and the ARI obtained setting the prior as specified on the x-axis labels.
