# Supplementary material for: A Bayesian method to infer copy number clones from single-cell RNA and ATAC sequencing
Source: PLoS Comput Biol. 2023 Nov 2;19(11):e1011557. doi: 10.1371/journal.pcbi.1011557 (PMC10645363; doi:10.1371/journal.pcbi.1011557)
Supplement: S2 Text — (PDF) [file pcbi.1011557.s002.pdf]

S2 Text

1 Extended materials and methods

The goal of CONGAS+ is that of identifying clusters of cells that are characterized by the same Copy Number Alterations (CNAs) over each segment. In what follow, we refer to total CNAs and not allele-specific copy number values, i.e., we do not distinguish major/minor allele counts, but just their sum, sometimes referred to as segment ploidy. The input segmentation can be:

- set to chromosome arms;
- set to a custom segmentation, such as one obtained from an independent bulk DNA sequencing assay applied to the same in put material.

The default fixed resolution is to determine arm-level CNAs. Either ways, given the segments, we use their coordinates to aggregate the RNA and ATAC signal of each single cell. This is done by summing count data (transcript or chromatin peaks) per segment. Working with segments makes it easier to detect CNAs. Whereas with bulk DNA assays we attempt a de novo segmentation of the tumour genome, here we considered that in the majority of cases every single-cell data is matched with a cheaper bulk assay, which can be used to generate a segmentation using standard bioinformatics tools (e.g., [9, 17, 18]). Using segments as baseline resolution allows also to assume independence of the pooled counts; we discuss model assumptions in Section 1.2

CONGAS+ models the observed counts for each single-cell in each segment as variables dependent on the (latent) copy number state of that segment. We assume that the hidden state influences the gene expression and chromatin accessibility signal through a linear relation. This is based on the intuition that, if one tumor cell loses one allele in a segment, the genes on that segment will have weaker signal compared to the genes off segment. This model has already been introduced in [7], and exploited in CONGAS, our previous work [13]. Here, we extended this idea to the chromatin accessibility signal, assuming that higher/lower copy number will increase/decrease the amount of open chromatin detected in the segment.

1.1 Full formulation of CONGAS+

The model takes in input two single cell datasets X^A and X^R of $N \in \mathbb{N}$ cells in the RNA sample and $M \in \mathbb{N}$ in the ATAC one, containing respectively the signal of RNA and ATAC over $I \in \mathbb{N}$ segments. These two matrices are the result of a pre-processing step, were the counts are aggregated over each segment i by summing all the features that map to i, with $i = 1, \dots I$. RNA features usually correspond to genes, while ATAC features are peaks or fixed-length genome bins. Both matrices can either be non-normalised integer counts ($\in \mathbb{N}_0^{N\times I}$) or normalised values ($\in \mathbb{R}^{N\times I}$), where in the latter case, we compute the z-score for

each input feature (see Section 1.1.2). The type of input provided (continuous versus discrete) determines the statistical distribution used to model the data.

CONGAS+ is a finite Dirichlet mixture of $K \geq 1$ distributions that model the K clones present in the single-cell samples. We first introduce a formulation as if we were to work with one single type of data, which allows to simplify notation by dropping the superscript that refers specifically to ATAC and RNA. Moreover, we consider the plain formulation of CONGAS+ where the input cells are independent.

The assignment of each cell to one of the $K \in \mathbb{N}$ mixture components is modeled by a categorical latent variable $\mathbf{z} = [z_{n,k}]$. The joint likelihood over the observed variables ($\mathbf{X}$) and latent variables ($\mathbf{z}$) of our model is

$$p(\mathbf{X}, \mathbf{z}|\boldsymbol{\theta}, \boldsymbol{\pi}, \boldsymbol{\Phi}) = p(\mathbf{z}|\boldsymbol{\pi})p(\mathbf{X}|\mathbf{z}, \boldsymbol{\theta}, \boldsymbol{\Phi}) = \prod_{n=1}^{N}\prod_{k=1}^{K} \boldsymbol{\pi}_{\boldsymbol{k}}^{z_{n,k}} \prod_{i=1}^{I} f(x_{n,i}, \boldsymbol{\theta_i}, \boldsymbol{\Phi})^{z_{n,k}} \quad (1)$$

where

$$p(\mathbf{z}) = \prod_{n=1}^{N}\prod_{k=1}^{K} \pi_k^{z_{n,k}}$$

is the distribution of the cell assignments, and $p(\mathbf{X}|\mathbf{z}, \boldsymbol{\theta}, \boldsymbol{\pi}, \boldsymbol{\Phi})$ is the conditional distribution of the observed variables given a specific cell assignment $\mathbf{z}$

Here f is a generic likelihood function that models the observed signal for the data, π are the clusters mixing proportions and Φ is a $K \times I \times H$ tensor for the probability distribution over discrete CNAs for each cluster and segment. In fact, each of the k clusters is associated to a probability distribution per segment $\phi_{k,i,h} = P(C_{k,i} = h)$ over the possible copy number values $h = 1, .., H$ that the $i - th$ segment may assume, where by default, $H = 5$.

This is the general formulation for a single omics, in the general setting we will have some parameters that are shared across data types and some others that are specific. In particular, the two likelihood for RNA (R) and ATAC (A) are obtained by marginalizing $\mathbf{z}$ in equation (1)

$$p(\mathbf{X}^A|\boldsymbol{\theta^A}, \boldsymbol{\pi}, \boldsymbol{\Phi}) = \sum_{\mathbf{z}^A} p(\mathbf{X}^A, \mathbf{z}^A|\boldsymbol{\theta^A}, \boldsymbol{\pi}, \boldsymbol{\Phi}) = \prod_{n=1}^{M}\sum_{k=1}^{K} \boldsymbol{\pi_k} \prod_{i=1}^{I} f(x_{n,i}^A|\boldsymbol{\theta_i^A}, \boldsymbol{\Phi}) \quad (2)$$

$$p(\mathbf{X}^R|\boldsymbol{\theta^R}, \boldsymbol{\pi}, \boldsymbol{\Phi}) = \sum_{\mathbf{z}^R} p(\mathbf{X}^R, \mathbf{z}^R|\boldsymbol{\theta^R}, \boldsymbol{\pi}, \boldsymbol{\Phi}) = \prod_{n=1}^{N}\sum_{k=1}^{K} \boldsymbol{\pi_k} \prod_{i=1}^{I} f(x_{n,i}^R|\boldsymbol{\theta_i^R}, \boldsymbol{\Phi})$$

so that the overall likelihood is a linear combination of the single ones terms

$$p(\mathbf{X}|\boldsymbol{\theta}, \boldsymbol{\pi}, \boldsymbol{\Phi}) = \lambda \cdot p(\boldsymbol{X}^{\mathrm{R}}|\underbrace{\boldsymbol{\theta}^{\mathrm{R}}, \boldsymbol{\pi}^{\mathrm{R}},}_{\Omega_{\mathrm{R}}} \boldsymbol{\Phi}) + (1 - \lambda) \cdot p(\boldsymbol{X}^{\mathrm{A}}|\underbrace{\boldsymbol{\theta}^{\mathrm{A}}, \boldsymbol{\pi}^{\mathrm{A}},}_{\Omega_{\mathrm{A}}} \boldsymbol{\Phi}) \quad (3)$$

where λ is a shrinkage hyperparamer.

For the sake of readability, we sometimes drop the data-type R/A index when not strictly necessary; the reader can refer to the probabilistic graphical

models reported in the Main Text for a complete view of the joint distributions and their factorisation. Moreover, we note that the multiomics assay is a simple special case of this model, where the latent variables are shared across RNA and ATAC cells.

1.1.1 Dirichlet priors for cluster and CNAs

The prior on $\mathbf{\Phi}$ is a Dirichlet distribution

$$\Phi_{k,i,h} \sim \text{Dirichlet}(\alpha), \qquad \alpha = (\alpha_1, ..., \alpha_K), \;\; \alpha_i \in \mathbb{R}_{>0}, \tag{4}$$

where the concentration vector α is a hyperparameter chosen by the user. Note that the tensor Φ does not change between modalities since cells from both omics are assigned to the same set of clusters.

CONGAS+ accommodates settings where the two data types have clusters in different proportions, which is achieved by using two different vectors π^{atac} and π^{rna}, that model the mixing proportion for each cluster. Each entry π_k is linked to another Dirichlet distribution

$$\pi_k^R \sim \text{Dirichlet}(\nu^R), \qquad \pi_k^A \sim \text{Dirichlet}(\nu^A) \tag{5}$$

where we choose $\nu^R = \nu^A = (1/K, ..., 1/K)$. The model allows also a special formulation where a shared parameter π controls the mixing proportions in ATAC and RNA jointly – this allows to have clusters with the same proportions in both RNA and ATAC, even if cells are independent.

1.1.2 Likelihood functions

The generic likelihood function f in equation eq. (2) is defined based on the type of input matrix provided. On the one hand, for integer count matrices we use a Negative Binomial (NB) distribution to model the observed signal. While, with normalised counts, each feature is z-scored prior to aggregating the signal over each segment, and f is defined as a Gaussian likelihood.

Integer counts. With segment-specific integer counts we use the Negative Binomial likelihood,

$$f_k(x_{n,i}|\boldsymbol{\theta_i}, \boldsymbol{\Phi_{k,i}}) = \text{NegBin}\left(x_{n,i}\middle|\frac{\mu_{k,i,n}}{\mu_{k,i,n}+r_i}, r_i\right) \tag{6}$$

where $\mu_{k,i,n}$ and r_i are the mean and size of the density. The prior distribution on r_i is

$$r_i \sim \text{Unif}(a_i, b_i)$$

for some choice of the extremes a_i, b_i. The mean of the density is defined as

$$\mu_{k,i,n} = \underbrace{(\rho_n \cdot \theta_i)}_{\text{Normalisation}} \cdot \underbrace{\left(\sum_h \Phi_{k,i,h} \cdot h\right)}_{\text{CNA mixture}} \tag{7}$$

Here θ_i are omic-specific variables that represent the average signal of a single copy of the i-th segment. For these quantities we choose a Gamma prior

$$\theta_i \sim \Gamma(\alpha_i, \beta_i), \tag{8}$$

where the hyperparameters α_i, β_i can be estimated from the data. To do so, in the `Rcongas` package we provide function `auto_config_run` to learn the hyperparameters values from segment counts, using a Maximum Likelihood Estimation (MLE) optimization process. The function uses the R package `fitdistrplus` [8] and an input bulk ploidy provided by the user when creating the CONAGS+ object (if missing, it assumes diploid).

By using a Negative Binomial with CNA-scaled mean we are modeling a signal for each cluster/ segment that is proportional to the number of DNA copies. In fact, the distribution $\Phi_{k,i}$ models the probability to detect each CNA value $h \in 1, \dots, H$ (by default $H = 5$) for each cluster k and segment i, and thus the mean of the density depends on the linear combination (dot product) of the latent CNAs.

Note that we also introduce cell-specific normalization factors ρ_n, which take into account possible expression differences due to sequencing. These are hyperparameters of the model and can be estimated from the data. The user can provide custom normalization factors as input; in general, those can be some function of the total number of counts by cell or more sophisticated estimates such as those computed by specialized tools like SCRAN [12]. If not provided, CONGAS+ automatically calculates normalizing factors by summing all the counts for a given cell, and scales the value for 10^x where x is the median number of digits. For instance, for numbers of the order of 10^3, it will re-scale the library size by a factor of 1000, re-scaling all values to be around 1. For all our examples in the paper, we used this latter method.

Normalized counts. CONGAS+ supports also datasets where prior to aggregation, each feature has been z-scored. In this case we assume the aggregated signal over each segment to be normally distributed

$$f_k(x_{n,i}|\boldsymbol{\theta_i}, \boldsymbol{\Phi_{k,i}}) \sim \mathcal{N}(\mu_{k,i}, \sigma_i) \tag{9}$$

where the mean equals the copy number value

$$\mu_{k,i} = \sum_h \phi_{k,i,h} \cdot h \tag{10}$$

and the standard deviation σ_i is sampled from a uniform distribution

$$\sigma_i \sim \text{Unif}(a_i, b_i). \tag{11}$$

The mean of the Gaussian presented in eq. (10) is analogous to that of the Negative Binomial in eq. (7): $\Phi_{k,i}$ is the distribution over possible discrete CNA values $h \in 1, \dots, H$ for each cluster k and segment i, and thus the mean of the Gaussian depends on the linear combination of the latent CNAs.

1.1.3 Informing CN inference with bulk sequencing

CONGAS+ has one optional parameter that can be used to penalize model solutions that are inconsistent with the input CNAs, when these are provided by orthogonal CNA calling from a bulk assay. In this case, we assume we are given also η, the bulk sample purity as obtained by copy number calling [2].

In this case, for each segment i where p_i is the input ploidy, CONGAS+ computes the penalization factor s_i

$$s_i = \left(\sum_k \pi_k \cdot \sum_h \phi_{k,i,h} \cdot h \right) - [p_i \cdot \eta + 2 \cdot (1 - \eta)] \tag{12}$$

where η is sample purity.

The total penalization is computed as

$$s = \sqrt{\sum_i (s_i)^2}$$

, and it is subtracted from the total likelihood $p(\mathbf{X}|\boldsymbol{\theta}, \boldsymbol{\pi}, \boldsymbol{\Phi})$. This factor s_i is a penalization for solutions that deviate from the input bulk p_i, and it can be naturally used in samples that are composed of admixed tumor and normal cells.

While this functionality is useful in principle, we did not use it in the three case studies reported in the main text. This because we either used cell lines (hence, samples composed of only tumor cells), and sample composed of admixed tumor and normal cells, but for which we did not have any information to determine p_i.

1.1.4 Parameters estimation

Our inference algorithm requires marginalizing the likelihood with respect to the clustering and copy number assignment, and calculating the Maximum A Posteriori (MAP) estimates of the continuous parameters. The parameters are learnt by stochastic variational inference.

Stochastic variational inference. We seek to estimate the posterior distribution for all parameters, using Bayes rule. For simplicity, we use U to indicate all the parameters in the model andwrite the posterior as

$$p(U|X) = \frac{p(X|U)p(U)}{p(X)} \tag{13}$$

where $p(X)$ is the marginal likelihood, also called evidence. The denominator is usually intractable, and we need to approximate the real posterior. CONGAS+, like its predecessor CONGAS, uses Stochastic Variational Inference (SVI) [15] to get the approximation of the true posterior $p(U|X)$. The aim of SVI is to find a variational distribution $q(U)$ that belongs to a family of probability

distributions $\mathcal{Q}$, which can approximate the real posterior. Learning in this setting is formulated as an optimization problem, where the goal is to minimize the Kullback-Leibler (KL) divergence between $p(U|X)$ and $q(U)$ [15, 6]

$$q^*(U) = \underset{q(U)\in\mathcal{Q}}{\arg\min}\{\mathrm{KL}[q(U)||p(U|X)]\} \tag{14}$$

However, this term is still untractable, as it requires to compute the posterior. Thus, the objective function that gets optimized in SVI is the Evidence Lower Bound (ELBO)

$$\mathrm{ELBO}(q) = \mathbb{E}\left[\log p(U, X)\right] - \mathbb{E}\left[\log q(U)\right]. \tag{15}$$

and maximizing this quantity is equivalent to minimizing the KL divergence [11, 5, 15]. In our case the variational distribution q is specified by a set of parameters γ – which acts as a placeholder for all the parameters we want to estimate, so in our specific case $\gamma = [\boldsymbol{\theta}, \boldsymbol{\pi}, \boldsymbol{\Phi}, \boldsymbol{z}]$. We learn γ during the inference, and in order to optimize the ELBO, SVI computes gradient descent optimization taking a Monte Carlo estimates of the gradient

$$\nabla\gamma\mathrm{ELBO} = \nabla_\gamma\mathbb{E}_{q_\gamma(U)}\left[\log p(x, u) - \log q_\gamma(U)\right]. \tag{16}$$

CONGAS+ is implemented in `Pyro` [4], a probabilistic programming language based on `Python` which implements SVI – we use Adam as numerical optimizer, which is appropriate for gradients that are very noisy or sparse.

Gumbel-Softmax to model categorical distributions within SVI. We use SVI to approximate the posterior via gradient descent. However, our model contains a discrete random variable Φ, for the probability distribution over the possible discrete copy number values. Categoricals are difficult to manage in a gradient-based optimisation as they create discontinuities in samples.

In order to be able to estimate the gradient for this categorical, we use the Gumbel-Softmax [10], a continuous distribution that can approximate samples from a categorical distribution. Considering the categorical Φ – which has H probability classes $\alpha_1, \alpha_2, \ldots, \alpha_H$ – to model the distribution over the possible copy number values, a sample ω from such distribution can be seen as a one-hot vector that lies on the corners of a $(h-1)$-dimensional simplex Δ^{h-1}

$$\omega = \mathrm{one\_hot}\Big[\mathrm{argmax}_h(g_h + \log\alpha_h)\Big] \tag{17}$$

where

$$g_h \sim \mathrm{Gumbel}(0, 1).$$

The Gumbel density function is often used in extreme value theory to model the distribution of the maximum or minimum of a large number of random variables; its density function is

$$f(x; \mu, \beta) = \frac{1}{\beta}\exp\left[-\frac{x-\mu}{\beta} - \exp\left(-\frac{x-\mu}{\beta}\right)\right]$$

In order to approximate the argmax and make everything continuous and differentiable, the softmax is employed. Thus, a H-dimensional sample vector from the Gumbel-Softmax distribution is a vector $\omega \in \Delta^{h-1}$ defined as

$$\omega_h = \frac{\exp\left[(\log \alpha_h + g_h)/\tau\right]}{\sum_{j=1}^{k} \exp\left[(\log \alpha_j + g_j)/\tau\right]}$$

where $i = 1, 2, \ldots k$ and τ is a temperature parameter. As τ approaches 0, the samples from the Gumbel-Softmax become one-hot vectors and thus sampling becomes identical to drawing samples from the categorical distribution Φ.

In the Gumbel-Softmax definition, α is the vector of parameters of the distribution, and it corresponds to the vector of the probabilities for the H classes of the categorical distribution. For values of the temperature greater than zero, the Gumbel-Softmax has a well defined gradient with respect to its parameters, and thus if we replace the categorical samples with the Gumbel Softmax it is possible to use backpropagation during training to compute the gradients.

However, the samples from the Gumbel-Softmax are not identical to samples from the corresponding categorical distribution when τ is not zero and thus there is the need for identifying a trade-off between large and small temperatures. In fact, on the one hand for temperatures close to zero samples are close to one-hot, but the variance of the gradients is large. On the other hand, large temperatures yield small gradient variance but smooth samples. The solution is to decrease the temperature followig a schedule: in Pyro we start from a value τ_{start}, and then at each step j of gradient descent optimization we update the temperature following this iterative formula

$$\tau_j = \tau_{start} / \log(j + 0.1)\,.$$

Computation of CNAs and clustering assignments. Once the MAP estimators have been computed, we can compute the copy number profile of each cluster $C_{k,i}$ by taking

$$C_{k,i} = \arg\max_h \left(\phi_{k,i,h}\right).$$

Given the copy number states, we compute clustering assignment probabilities $P^t_{n,k}$ of the cells for both modalities. These are

$$P_{n,k} = \frac{\pi_k \prod_i f\left(x_{n,i} | \Phi_{k,i}, \theta, \rho\right)}{\sum_k \pi_k \prod_i \left(f\left(x_{n,i} | \Phi_{k,i}, \theta, \rho\right)\right)} \tag{18}$$

and using the above probabilities we estimate for each cell the assignment vector $z_{n,k}$

$$z_{n,k} = \begin{cases} 1 \text{ if } k = \text{argmax}\left(P_{n,k}\right) \\ 0 \text{ otherwise} \end{cases} \tag{19}$$

1.2 Statistical assumptions and limitations of the model

To make the inference of copy numbers computationally fast and mathematically efficient, the model makes certain statistical assumptions which, in turn, translate into biological assumptions.

Data types. The main assumption concerning the integration of the two data modalities is that the subclonal structure of the RNA and ATAC samples is the same. While this is always valid in the case of multiomics single-cell experiments, with independent assays this could not be valid.

Precisely, while this should be generally valid when both ATAC/ RNA are processed from the same starting material, the same might not hold if samples come from different spatial regions of the tumour. In that case, the risk of having little overlap between clonal populations is higher.

Likelihood. Most of the assumptions on the actual likelihood are shared with the original CONGAS [13], and extended to ATAC where the original model was limited to RNA.

In particular these assumptions are:

1. The presence of a linear relation between the number of copies of a segment of DNA, and the average expression of the genes mapping in that segment (for RNA) and Tn5 read coverage (for ATAC);
2. Given a segmentation, all the segments are conditionally independent given the copy number values;
3. The segmentation is correct for all the cells in our samples;
4. Copy number variation is the main biological driver of expression variance in a segment when considering all the genes, or when considering all the peaks (open chromatin regions).

We believe assumption 1 to be extremely reasonable for ATAC because this assay directly captures DNA. For RNA, since expression is much more complex and involves a complex regulatory network and concerted pathway behaviors, this might be more complicated. To control this we introduce the hyper-parameter λ, see S3 Text. We partially study the model behavior when assumption 2 is not valid in 2.2, finding that overall we can still get informative results.

The last assumption is the most critical one, and highlights the importance of having good pre-processing of the data to avoid possible biological and technical biases as alternative explanations of the data. Of course, our idea is that CNAs inferred by CONGAS+ should be investigated to try to rule out possible locus-specific biological processes not related to copy number alterations.

2 Additional details on case studies

2.1 Basal Cell Carcinoma (BCC) for hyperparameter tuning

We collected data from [21] and [16] where the authors performed scRNA-seq, Whole Exome sequencing (WXS) and scATAC-seq on samples collected from patients affected by Basal Cell Carcinoma (BCC). Patients underwent treatment against BCC, and sequencing experiments were performed both pre and post treatment.

The datasets consist of both tumor and normal cells, and in order to assess the ability of CONGAS+ in identifying CNA-associated clusters with varying values of λ, we considered the single-cell labeling provided by the original authors. To make this test, we restricted to those datasets for which there was a significant presence of both tumor and normal cells.

First, we analyzed patients SU006 and SU008 where all three assays were performed on both tumor and normal tissues. We used this to see if CONGAS+ can distinguish tumor from healthy diploid cells. We selected an equal number of tumor and normal cells to build a dataset for illustrating the performance for varying values of hyperparameter λ. For bulk segmentation, we downloaded FASTQ files of both tumor and normal cells from SRA and we applied GATK [18] best practices for alignment: we performed alignment with `bwa-mem`, we removed duplicates with Picard and we recalibrated base quality scores. Finally, we used the obtained BAM files as input for Sequenza [9] to identify CNA profiles to then use with CONGAS+.

2.2 Chromosome arm segmentation on a Gastric cell line dataset

We collected scDNA-seq and scRNA-seq from [1], and scATAC-seq from [20] for gastric cancer cell line (SNU601). In [20], from 10 copy number segments 6 subclones (labeled 1 to 6) were determined by using scDNA-seq, and matched against scATAC-seq data. From these subclones, we merged clones 3 and 6 (still labeled 6) that were associated with segments with less than 5 genes because these are too few to be used with CONGAS+.

We ran CONGAS+ on scATAC-seq and scRNA-seq using chromosome arm segmentation and diploid priors. Our analysis ($\lambda = 0.5$, scored by BIC) identified 3 subclones which we used to verify that CONGAS+ infers the correct integer copy number values for each clone at the chromosome arm resolution. We computed the overlap between each CONGAS+ cluster and scDNA-seq clone and observed the majority of cells of three clones were assigned to one cluster, while the other two clones were subdivided between two clusters (Fig.S1A). The same cluster-clone correspondence was obtained by the mean absolute deviation between the integer copy number profiles (FigS1B). Using chromosome arm resolution, we could infer only subclonal copy number involving segments which had a high overlap with the arm in which they were contained (FigS1C,D,E).

An example is chromosome 11q, for which the scDNA-seq segment coincided with the entire arm. On the other hand, the segment contained in 1q had an overlap of 0.25 with the arm and CONGAS+ was not able to detect the expected amplification. We reported in FigS1F,G,H the normalised RNA counts for chromosome 1q and 11q at chromosome arm and scDNA resolution. While for chr11q the two resolutions were equivalent and we observed a bimodal distribution, the distributions changed with the resolution in case of chromosome 1q. Considering the entire arm we could not appreciate differences between scDNA-seq clones, while at scDNA-seq resolution clone 1 showed to have a lower peak of counts distribution. This behaviour explained why CONGAS+ could not infer the subclonal copy number using chromosome arm segmentation.

References

[1] Noemi Andor, Billy T Lau, Claudia Catalanotti, Anuja Sathe, Matthew Kubit, Jiamin Chen, Cristina Blaj, Athena Cherry, Charles D Bangs, Susan M Grimes, et al. Joint single cell dna-seq and rna-seq of gastric cancer cell lines reveals rules of in vitro evolution. *NAR Genomics and Bioinformatics*, 2(2):lqaa016, 2020.

[2] Alice Antonello, Riccardo Bergamin, Nicola Calonaci, Jacob Househam, Salvatore Milite, Marc J Williams, Fabio Anselmi, Alberto d'Onofrio, Vasavi Sundaram, Alona Sosinsky, William CH Cross, and Giulio Caravagna. Computational validation of clonal and subclonal copy number alterations from bulk tumour sequencing. *bioRxiv*, 2023.

[3] Giacomo Baruzzo, Ilaria Patuzzi, and Barbara Di Camillo. SPARSim single cell: a count data simulator for scRNA-seq data. *Bioinformatics*, 36(5):1468–1475, 10 2019.

[4] Eli Bingham, Jonathan P. Chen, Martin Jankowiak, Fritz Obermeyer, Neeraj Pradhan, Theofanis Karaletsos, Rohit Singh, Paul A. Szerlip, Paul Horsfall, and Noah D. Goodman. Pyro: Deep universal probabilistic programming. *J. Mach. Learn. Res.*, 20:28:1–28:6, 2019.

[5] Christopher M Bishop and Nasser M Nasrabadi. *Pattern recognition and machine learning*, volume 4. Springer, 2006.

[6] David M Blei, Alp Kucukelbir, and Jon D McAuliffe. Variational inference: a review for statisticians. arxiv. *arXiv preprint arXiv:1601.00670*, 2016.

[7] Kieran R Campbell, Adi Steif, Emma Laks, Hans Zahn, Daniel Lai, Andrew McPherson, Hossein Farahani, Farhia Kabeer, Ciara O'Flanagan, Justina Biele, et al. clonealign: statistical integration of independent single-cell rna and dna sequencing data from human cancers. *Genome biology*, 20(1):1–12, 2019.

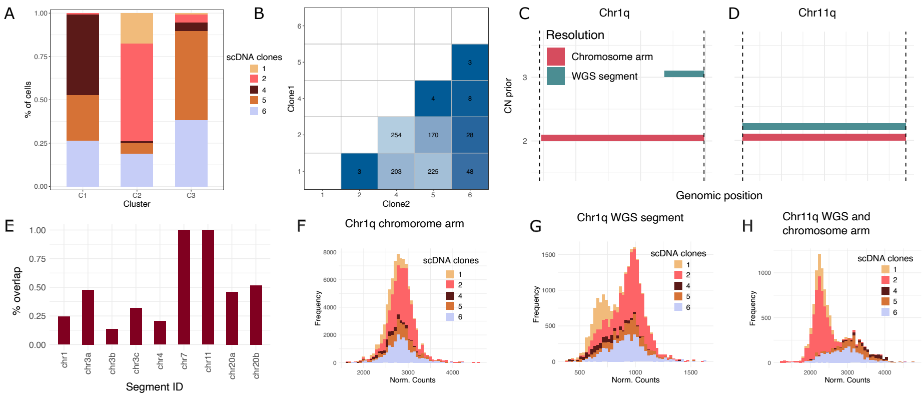

Figure S1: **Gastric cell line at chromosome arm resolution A.** Percentages of scDNA clones mapped on clusters inferred by CONGAS+ with chromosome arm resolution. We observed that cells of clones 1 and 2 were mostly assigned to cluster C2, clone 4 was mainly contained in C1, clone 5 was spread accross C1 and C3, while clone 6 overlapped with all clusters. **B.** Number of differentially expressed peaks between scDNA clones. Our analysis revealed that clones 1,2 and clones 4,5,6 had very few differentially expressed peaks and therefore a similar trascriptional profile. On the other hand, these two groups showed a high number of differentially expressed peaks against each other. **C-D.** Copy number priors that we used in CONGAS+ inference at chromosome arm resolution (red) and WGS segment resolution (green). For Chr11q we adopted a diploid prior for both resolutions, while in the case of Chr1q we used a triploid prior for the WGS segment. **E.** Overlap of WGS segments with the chromosome arms containing them. In case of chr7 and chr11 the entire arm is covered, while for chr2, chr20a, chr20b the segment overlapped with half of the arm. **F-G-H.** Normalized RNA counts mapped on chromosome 1q and 11q for scDNA clones at resolution of chromosome arm and WGS. In case of chr11q the two resolutions are equivalent and we observed a bimodal distribution which split scDNA clones in two groups, containing respectively clones 1,2 and clones 4,5,6. On the other hand, the distributions changed with the resolution in case of chromosome 1q. Considering the entire arm we cound not appreciate differences between scDNA clones, while at WGS resolution clone 1 showed to have a lower peak of counts distribution.

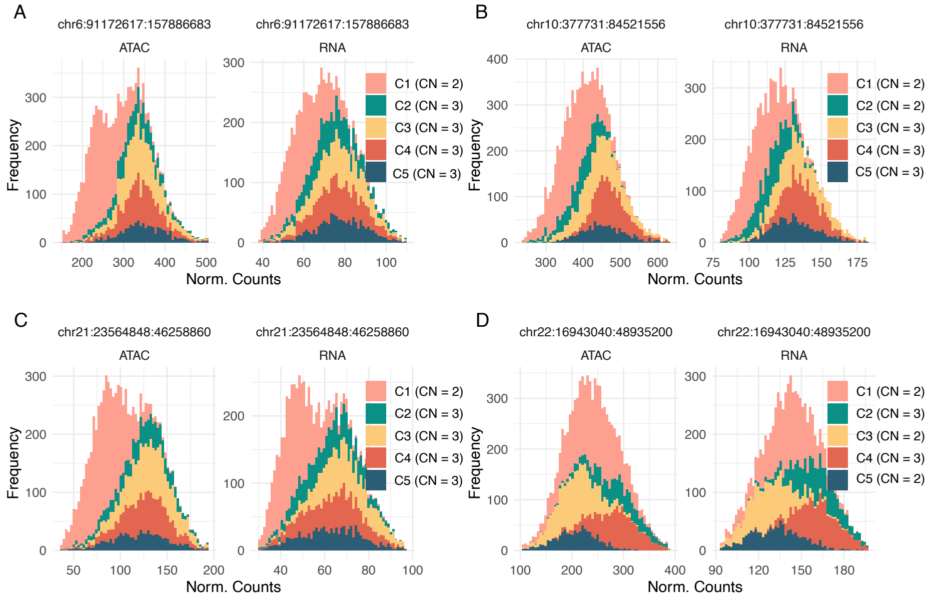

Figure S2: **Counts distribution for CNA segments in the Prostate cancer cell line.** **A,C.** two copy number events that are shared among all the resistant cells. **B** amplification detected in cells from C3, C4 and C5. **D.** segment where there is one subclonal copy number event that distinguishes cells in C2 and C4 from the other clusters.

[8] Marie Laure Delignette-Muller and Christophe Dutang. fitdistrplus: An R package for fitting distributions. *Journal of Statistical Software*, 64(4):1–34, 2015.

[9] Francesco Favero, Tejal Joshi, Andrea Marion Marquard, Nicolai Juul Birkbak, Marcin Krzystanek, Qiyuan Li, Z Szallasi, and Aron Charles Eklund. Sequenza: allele-specific copy number and mutation profiles from tumor sequencing data. *Annals of Oncology*, 26(1):64–70, 2015.

[10] Eric Jang, Shixiang Gu, and Ben Poole. Categorical reparameterization with gumbel-softmax. *arXiv preprint arXiv:1611.01144*, 2016.

[11] Michael I Jordan, Zoubin Ghahramani, Tommi S Jaakkola, and Lawrence K Saul. An introduction to variational methods for graphical models. *Machine learning*, 37(2):183–233, 1999.

[12] Aaron T L Lun, Karsten Bach, and John C Marioni. Pooling across cells to normalize single-cell rna sequencing data with many zero counts. *Genome biology*, 17(1):1–14, 2016.

[13] Salvatore Milite, Riccardo Bergamin, Lucrezia Patruno, Nicola Calonaci, and Giulio Caravagna. A bayesian method to cluster single-cell rna sequencing data using copy number alterations. *Bioinformatics*, 38(9):2512–2518, 2022.

[14] Zeinab Navidi, Lin Zhang, and Bo Wang. simatac: a single-cell atac-seq simulation framework. *Genome biology*, 22(1):1–16, 2021.

[15] Rajesh Ranganath, Sean Gerrish, and David M Blei. Black box variational inference. *arXiv preprint arXiv:1401.0118*, 2013.

[16] Ansuman T Satpathy, Jeffrey M Granja, Kathryn E Yost, Yanyan Qi, Francesca Meschi, Geoffrey P McDermott, Brett N Olsen, Maxwell R Mumbach, Sarah E Pierce, M Ryan Corces, et al. Massively parallel single-cell chromatin landscapes of human immune cell development and intratumoral t cell exhaustion. *Nature biotechnology*, 37(8):925–936, 2019.

[17] Eric Talevich, A Hunter Shain, Thomas Botton, and Boris C Bastian. Cnvkit: genome-wide copy number detection and visualization from targeted dna sequencing. *PLoS computational biology*, 12(4):e1004873, 2016.

[18] Geraldine A Van der Auwera and Brian D O'Connor. *Genomics in the cloud: using Docker, GATK, and WDL in Terra*. O'Reilly Media, 2020.

[19] Chenfei Wang, Dongqing Sun, Xin Huang, Changxin Wan, Ziyi Li, Ya Han, Qian Qin, Jingyu Fan, Xintao Qiu, Yingtian Xie, et al. Integrative analyses of single-cell transcriptome and regulome using maestro. *Genome biology*, 21(1):1–28, 2020.

[20] Chi-Yun Wu, Billy T Lau, Heon Seok Kim, Anuja Sathe, Susan M Grimes, Hanlee P Ji, and Nancy R Zhang. Integrative single-cell analysis of allele-specific copy number alterations and chromatin accessibility in cancer. *Nature biotechnology*, 39(10):1259–1269, 2021.

[21] Kathryn E Yost, Ansuman T Satpathy, Daniel K Wells, Yanyan Qi, Chunlin Wang, Robin Kageyama, Katherine L McNamara, Jeffrey M Granja, Kavita Y Sarin, Ryanne A Brown, et al. Clonal replacement of tumor-specific t cells following pd-1 blockade. *Nature medicine*, 25(8):1251–1259, 2019.
