# Supplementary material for: A Bayesian method to infer copy number clones from single-cell RNA and ATAC sequencing
Source: PLoS Comput Biol. 2023 Nov 2;19(11):e1011557. doi: 10.1371/journal.pcbi.1011557 (PMC10645363; doi:10.1371/journal.pcbi.1011557)
Supplement: S3 Text — (PDF) [file pcbi.1011557.s003.pdf]

S3 Text

1 Additional details on simulations

1.1 RNA/ATAC for a normal population

In order to perform extensive simulations of CONGAS+, we have generated synthetic datasets. To obtain coupled scRNA-seq and scATAC-seq datasets, we employed two tools that simulate data by estimating parameters from real single-cell datasets, namely SPARSim [1] and simATAC [2]. The rationale to use external tools – instead of the generative model underneath CONGAS+ – is to avoid any bias in data generation that might favour our performance.

In detail, we downloaded two public datasets of human Peripheral Blood Mononuclear cells (PBMC) cells from the 10x genomics website

https://www.10xgenomics.com/resources/datasets

and after performing quality check and clustering through Seurat and Signac we associated a PBMC cell type to each cluster with the tool MAESTRO [3], which exploits known gene expression signatures to assign labels based on cluster markers. We selected monocytes and neutrophils from scRNA and scATAC respectively, and we used their cells to obtain simulation parameters and generate synthetic datasets via SPARsim and simATAC.

simATAC [2] takes in input a bin x cells matrix and uses the real values to estimate the distribution of following simulation parameters: library size, non-zero cell proportion in each bin and mean signal for each bin. After sampling the library size for each cell i and the bin mean for each bin j, it simulates counts c_{ij} sampling from a Poisson distribution, where the mean of the Poisson is the library size scaled by the bin mean. The final output of the simulation is a bin times cells matrix of an homogeneous population of cells.

SPARsim [1] takes in input a single cell gene expression matrix and exploits it to estimate the model parameters, which are the following: feature intensity (i.e., vector representing the average expression level of each gene), biological feature variability and library size.

1.2 Copy number clones

We then added CNAs to the simulated normal data, considering simulated scenarios with increasing complexity in terms of clone-composition. To simulate segment breakpoints we follow this procedure:

1. we first divide the length of each chromosome c by a parameter that encodes the approximate segment length (by default we set it to $1e8$), to obtain the maximum number of segments imax_{chr_c};

2. we then randomly sample an integer i_{chr_c} ranging from 1 to imax_{chr_c}, which corresponds to the effective number of segments;

3. finally, we break each chromosome in exactly $i_c hrc$ segments and we randomise the breakpoints by adding a value sampled from a distribution $\mathrm{Unif}(-a, a)$, where a is equal to 10% of the average segment length.

In order to simulate a clonal architecture, we generate a tree with K clones by iteratively attaching a new node to a randomly selected leaf in the tree. The aneuploidy profile of each clone $y_k = (y_{k,1}, \dots, y_{k,I})$ is simulated by taking the parent profile and changing the copy number values of D randomly selected segments. Through this procedure, each new node will have a Hamming distance from its parent equal to D. Once the clonal architecture has been computed, we divide the normal simulated cells in K clusters, sampling their proportions from a Dirichlet distribution $\mathrm{Dirichlet}(1/K, \dots, 1/K)$, and for each segment i and each cluster k we take all the features mapping to i and we multiply their value by $y_{k,i}/2$. We simulated datasets with K ranging from 2 to 10, for a total of 90 datasets.

References

[1] Giacomo Baruzzo, Ilaria Patuzzi, and Barbara Di Camillo. SPARSim single cell: a count data simulator for scRNA-seq data. *Bioinformatics*, 36(5):1468–1475, 10 2019.

[2] Zeinab Navidi, Lin Zhang, and Bo Wang. simatac: a single-cell atac-seq simulation framework. *Genome biology*, 22(1):1–16, 2021.

[3] Chenfei Wang, Dongqing Sun, Xin Huang, Changxin Wan, Ziyi Li, Ya Han, Qian Qin, Jingyu Fan, Xintao Qiu, Yingtian Xie, et al. Integrative analyses of single-cell transcriptome and regulome using maestro. *Genome biology*, 21(1):1–28, 2020.
