# Supplementary material for: A Bayesian method to infer copy number clones from single-cell RNA and ATAC sequencing
Source: PLoS Comput Biol. 2023 Nov 2;19(11):e1011557. doi: 10.1371/journal.pcbi.1011557 (PMC10645363; doi:10.1371/journal.pcbi.1011557)
Supplement: S4 Text — (PDF) [file pcbi.1011557.s004.pdf]

S4 Text

Tuning the hyperparameter λ

CONGAS+ includes an hyperparamter λ that shrinks the likelihoods of RNA and ATAC during the inference, which can be used to weigh unevenly the evidence of the data. We use λ in the Main Text to correct for uneven signal quality. However, we did not find any formal way to decide the best λ against data (including learning it from data), but we provide the user with the following guideline to converge to what we think is a reasonable value of λ

1. Initialize the CONGAS+ object and plot the total signal (i.e., the data) for every segment. This can be done with the functions of the `Rcongas` package. Perform an initial qualitative check of the data by inspecting the modes of the distributions, the attempt is to visually assess the presence or absence of multimodalities in the data, and inspect for the different dispersion among ATAC and RNA signals. Consider that since each segment is one-dimensional, visual inspection is a valid aid to this task;

2. Perform an initial fit using the default value

$$\lambda = 0.5$$

   which weights evenly the data types.

3. Use the `Rcongas` function `plot_fit(congas_object, 'density')` that plots the inferred density together with the count histogram for each segment. That function can be exploited to qualitatively assess if data supports CNAs in one or both data types, and in general if the inferred distributions fit well the data.

4. Use two other functions to verify the quality of the fits: `plot_fit(congas_object, 'posterior_CNA')` to get an heatmap with the model uncertainty over CNAs in each segment, and `plot_fit(congas_object, 'scores')`, that plots the model selection scores and the likelihood of both modalities for all values of K tested.

5. If both data types fit well against the model, the adopted λ can be considered acceptable. Otherwise, if only one of the two data types fits well against the model, repeat steps 3–5 tuning λ accordingly. For example, if ATAC – as it often happens – has a better signals than RNA, then tune λ to lower values to give more weight to ATAC.
