# Supplementary material for: A Bayesian method to infer copy number clones from single-cell RNA and ATAC sequencing
Source: PLoS Comput Biol. 2023 Nov 2;19(11):e1011557. doi: 10.1371/journal.pcbi.1011557 (PMC10645363; doi:10.1371/journal.pcbi.1011557)
Supplement: Attachment — Submitted filename: Response to reviewers.pdf [file pcbi.1011557.s005.pdf]

Response to reviewers

Reviewer 1

The paper introduced a method CONGAS+ which combines scRNA and scATAC-Seq data to infer tumor subclones represented by CNAs using a Bayesian framework. While similar approaches to combining multiomic datasets are becoming commonplace, there is novelty in the use here. It is not obvious that there would be any synergy between these two particular data sources in inferring copy number profiles, but the paper provides a clear rationale for why this is the case. This is fairly tested on both real and simulated data to show the practical advantages of combining the two data sources. The author shows the shrinkage effect and demonstrates different reliability or signal quality among both modalities, in addition to providing specific guidance about parameter selection. The method shows relatively robust performance compared to other methods which only rely on scRNA-Seq. There is a good demonstration on real data that the approach can lead to novel biological discovery. The appendix adds some useful detail on methods and additional results, but the paper stands by itself well enough. Overall the work has potential to be a useful tool for dissecting tumor subclones based on single cell sequencing data with multiple modalities, which are becoming increasingly popular and informative in cancer genomics.

We thank the reviewer for noting the novelty in the use of a genomics-inspired latent space to map both RNA and ATAC at the same time, and for providing a fair and accurate assessment of our contribution.

Major:

1. There might need to be more explanation of how you generate the simulated data. Since your model assumes that the scRNA and scATAC are related to the corresponding copy number state, your simulation of scRNA/scATAC-Seq and copy numbers seems to be separate and not correlated, while in reality, we would expect stronger dependence due to confounding factors independent of copy number.

We clarify our procedure for simulation of synthetic data.

1. We gathered real datasets to obtain simulation parameters and then simulated RNA and ATAC normal cell counts using simATAC and SPARsim. This step, based on tools external to CONGAS+, ensures that i) any molecule-specific bias should be generated according to the implementation of simATAC/SPARsim, and ii) simulated data has parameters – including those causally linked to biases – that resemble a real use-case of our algorithm
2. We then added copy numbers to simulated “normal” data under sampled segment breakpoints and a clonal architecture as a randomly sampled tree. CNAs were simulated following lineage relations and changing the child CNAs randomly, based on the parental copy number profile.
3. We generated clusters mixing proportions from a Dirichlet distribution with uniform concentration, and we assigned cells to clusters.
4. RNA and ATAC counts were mapped to the input segments, and rescaled to follow the copy number profile: i.e., amplified, in case of gain, or halved in case of deletion.

This follows the CONGAS+ assumption that counts are linearly correlated to the ploidy of the corresponding segment.

We reported this procedure in the Main text (lines 108-119), and more details are reported as supplementary material (Section 2.1).

2. More specification needed in the “shrinkage effect with Basal Cell Carcinoma data” part on page 6. The meaning of “a signal present in just ATAC” or “in both RNA and ATAC” is not clear.

We apologise that the terminology was not clear.

We defined a signal (RNA or ATAC) as “present” if the distribution associated matches, in terms of modes, the latent copy number configuration. For example, for a subclonal amplification, since we expect a subset of cells to have slightly more counts than the rest, then the distribution for the amplified segment would be bimodal if the signal was present. Otherwise, we would consider that absent.

We clarified this in the Main Text (lines 167-168 and line 174), specifying which samples exhibit a bimodal signal.

3. It would be useful to comment on resource usage, preferably with real data behind it to give an idea of time and memory needs, scaling, and whether users need any specialized hardware for the method to be practical.

This matter has been also raised by Reviewer 3. Please refer to Major point Number 6 in the response.

Minor:
1. The paper as a whole could use more thorough proofreading. There are some sentences that don’t make sense. I.e. page 3 “while for RNA transcript counts.” and p. 9 “one to copy-neutral losses”.
2. On page 4, the joint likelihood should be joint log likelihood since it is adding both terms up.
3. On p. 8 of the appendix, there is a placeholder for the source of some data “data and cell annotations from xxx” that needs to be filled in.

We apologise for the missing and wrong text. We fixed the sentences pointed out in each of the three points.

Reviewer 2

This manuscript introduces the CONGAS+ algorithm, which aims to integrate scRNA-seq and scATAC-seq data to identify somatic copy number alterations in the sequenced cells and thus map these cells to the corresponding tumor clone. While previous methods have been introduced to address this problem only using scRNA or by integrating prior information about the underlying copy number alterations, this is the first algorithm integrating both scRNA-seq

and scATAC-seq data. Based on this, the manuscript demonstrates that the integration significantly improves the results compared to using a single data type alone, using both simulated and different cancer datasets. As such, I think that the study would be of great interest for the RECOMB CCB community and it represts a good prototype work that tick all the boxes for RECOMB: strong biological motivation, a new problem formulation, a novel sophisticated algorithmic contribution, and demonstrated application.

We thank this reviewer for noting the novelty of CONGAS+, and its good fit to the RECOMB community.

I have a few questions and comments to consider:

- One very important but unclear point is a statement of all the underlying assumptions on which the model relies on. For example, there seems to be a scaling factor between observed signals (either scRNA or scATAC) and underlying copy numbers which is constant across all genomic bins/genes. However, this is only an approximation, which is often not true in expression data for example. This can be a good starting point anyway, given the complexity of the problem, but the assumption should be clearly reported.

We apologise that we did not clearly state model assumptions. Now (Supplementary Information Text, Section 1.2) we clearly state both statistical and biological assumptions of our model.

- Is the lymphoma dataset an example of where CONGAS+ failed since two of the identified groups of cells did not have different copy number alterations but were more likely driven by differences in chromatin accessibility? The main conclusion behind this story should be better clarified.

We thank the reviewer for pointing out the issue in our analysis, and we now added a feature in our model that can be used to model normal cells in the data. Since we developed a Bayesian model, we do this by initialising one of the clusters in the data to be diploid, initialising the concentration parameter of the Dirichlet over CNAs in the following way:

Dirichlet(0.025, 0.9, 0.025, 0.025, 0.025)

so that more mass is given to the diploid state (0.9 is for the diploid state). This modification can aid in the discovery of normal cells in the data. We used this in the analysis of the multiome case study, whose results are described in the results section *"Tumour-normal deconvolution from ATAC/RNA multiomics"*, and in Figure 6 in the manuscript.

- I found the text to be quite technical and not easily accessible by a broader audience. I think that the introduction would benefit by adding more definitions and biological context (for example, defining tumour clones, copy number alterations, etc.), as well as each of the result sections would benefit from a introductory paragraph clearly describing the goal of the analysis and the details of the dataset analysed. For example, I found the section about the basal dataset to be particularly difficult to follow and lacking details about the related data. Also, most of the reasoning about the choice of the algorithmic technique could be moved to a related section.

We apologise that the writing was not clear. In this re-submission, we have rewritten almost all the Main Text, in particular we have

- Shortened and polished the Introduction and Conclusions, avoiding unnecessary jargon and focusing on the main message of the paper;
- Simplified the mathematical definition of the model in the Main Text, also providing more comments on the key aspects of the model;
- Focused the discussion in each case study, which should allow the broader readership understanding the rationale under each analysis and conclusion.
- Polished the description of the basal cell carcinoma dataset, to address also to point 2 raised by reviewer 1.

- I could not find the figures demonstrating the results showing that CONGAS+ using both scRNA-seq and scATAC-seq outperforms CONGAS+ using only either of the two. For example, CONGAS+ using both signals seem to be missing from Figure 1L. Also, I found Figure 1L and the other panels of the same figure difficult to interpret (I would make figures bigger, show the underlying data, etc.).

We split the original figure 1 in four pieces, to better organise the information. In detail, in Figure 3, we present an example of the simulated data, showing the segmentation and the counts distribution. We also separated all performance plots in Figure 4, and we made it clearer in panel 4E that CONGAS+ is run taking both omics as input.

- I could not find the details of how copy number alterations were added into the simulations; please include the details and related assumptions.

Please see Major point 1 of Reviewer 1.

- Why do you leave the choice between BIC and AIC to the user? The choice between BIC and AIC should be based on the difference between the numbers of parameters vs number of observations. In this context, the number of data points seem to be orders of magnitude higher than the number of parameters, motivating the choice of BIC. I would recommend to include this motivation rather than leaving the choice arbitrary.

We thank the reviewer for this observation, which we now use as motivation for our BIC choice. We included in the main text the explanation behind the choice of BIC as default value (lines 102-103), and we also leave the option to choose the model selection score for customisation reasons and backward compatibility.

- Why and how the values of Dirichlet parameters have been chosen to be 0.6 and 0.1, and how much does this choice impact the results? Importantly, how would the results in lymphoma change by varying the prior on ploidy/copy numbers? Maybe I prior higher for higher values of ploidies (B-cell lymphomas can be aneuploid) could provide a better fit and results for CONGAS+.

We chose the default prior based on the hypothesis that the tumour populations originate from a non-tumor population of normal cells with a heterozygous diploid genome, so that

higher prior probability should be assigned to diploid states. A strong enough signal of aneuploidy from the tumour cells can then push the likelihood to shift the expected probabilities to a reasonable posterior identifying aneuploid populations.

We exploited synthetic data to investigate in depth how CONGAS+ inference changes with respect to modifications to this prior probability, by measuring how the ARI between ground truth labels and inferred clusters changes after modifications to the prior. We now added a figure and the corresponding discussion in Section 2.2 of the supplementary information, showing that overall CONGAS+ is stable with respect to changes in the Dirichlet prior.

- Please define the domain of every variable introduced in the model (which are integers, real values, etc.).

We apologise for neglecting this important aspect of the mathematical formulation of our model. We specified the domain of every variable in the main text.

- The story in the prostate cancer data would benefit and be more convincing if you could show the raw data that support CONGAS+ findings, for example show the profiles of the cells that have been found in different groups.

We thank the reviewer for this comment. Given the presence of multiple cell lines merged together in the data, we decided to assess whether we could find subclones in the data. We now rerun the analysis, increasing the maximum number of clusters to be considered for the model selection procedure. From this rerun we have identified 5 clusters, which are now commented in detail in the manuscript. To make the story more convincing and analyse differences between inferred clusters, we have presented the underlying data by showing the count distributions for all cells on segments affected by copy number events, and we have carried out differential expression testing to analyse differences between the identified clusters. All details are in the Results section, in the paragraph entitled *"Drug-resistance associated with copy number subclones."*

- Could you please explain what reparametrization is and why is it needed?

We have added the domain for each variable. Moreover we added a short part about the reparameterization and why it is helpful in the Main Text from line 100. In brief, it allows to achieve a differentiable approximation to sampling discrete data.

- From the introduction, it is not clear that CONGAS+ infers copy number alterations; I would clarify it. Also, it is not immediately obvious that cancer is the key application of CONGAS+.

We have rewritten the full Introduction, avoiding unnecessary jargon and focusing on the main message of the paper, which include the possibility of inferring CNAs in the context of cancer single-cell studies.

Reviewer 3

This is an interesting paper, with an idea that seems to naturally "click" when introduced. Namely, the Authors solve a problem of joint clustering of cells by their copy-number profiles

into what they call CAN clones and identify the total copy number calls for segments in these clones. To do that, they integrate scRNA-seq with ATAC-seq. The paper is for the most part well written (although very short and some important details are missing) and the problem is well introduced. Unfortunately, neither the model presentation nor the results are convincing.

We thank this reviewer for finding our work appealing. In the new version we have largely improved the presentation, providing more convincing evidence of the model and results. We thank the reviewer in motivating us to improve the work that now seems more solid and better communicated.

1) Mathematical formulation and presentation should be improved:

- The main text contains some of the model formulation, which is to a large extent copied in the supplement 1.2, but there an additional index is added (t). Moreover, a mysterious \Delta appears in the equations in the supplement, which is nowhere introduced. I suggest cleaning up the math and the explanation, and keeping one version of model specification, to avoid differences between the two, which are highly confusing to the reader.

We thank the reviewer for pointing out inconsistencies in the mathematical formulation presented between the main and supplementary text. The use of \Delta was a typo for the correct name of the model parameters \theta. We replaced the correct term across the supplementary material. Regarding the index (t) that distinguishes RNA from ATAC modality, we removed it also in the supplementary text for consistency, specifying in a better way when some equations are modality specific and when not.

In the Main Text we specified the simplification at lines 78-79 by writing that “The generic CONGAS+ likelihood (for either RNA or ATAC [...]) is defined as [...])”.

- The plate presentation of the model is not correct. If some variables are shared, they cannot be copied twice in the representation, they should appear once. For example, variable v – it is only represented as if it was indexed by I, and occurs twice. The same for \pi. Also a minor point - the prior value for \pi on the right hand side (red \nu) is shifted to the left. It seems also that z is not valid. Why does X depend on z? In the formulation of the model (neither in the main nor in the supp), X does not depend on z.

We apologise that our graphical model was not clear. Our idea was to use colours (blue for RNA, red for ATAC) to denote that the variables are specific to the data type. This was supposed to be a shorthand to avoid using indexes over variables. Therefore in the previous version of the model the two variables v are different as if they were v_{RNA} and v_{ATAC}.

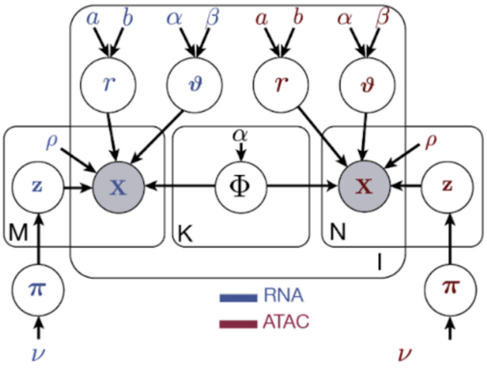

In order to avoid this confusion we have re-drawn the model in a more canonical way that reports all relevant indexes, on top of the colours. Variables that are shared across the data types (eg, copy number profiles) are in black. For example

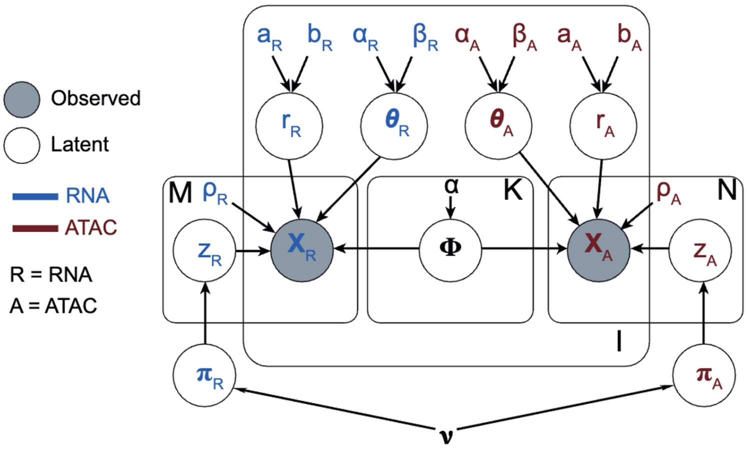

Regarding the dependency of X on z and its representation in the graphical model, we added a definition of the joint likelihood in the supplementary material (supplementary eq. 1) that explicits the dependency X on z. In the main text we explained that the likelihood presented in eq. 1 is obtained "after marginalising for the local latent variable z" (line 79).

2) Many details of the results are missing and the description is very short. For example, for the simulated data, was the true parameter K given to the model, or was the BIC used to find it? Also, what was the\Lambda value and why? Call commands of all tools used should be presented (can be in the supplement).

We thank the reviewer for pointing out the absence of these details. We analysed each simulated dataset using BIC to select the best number of clusters and a shrinkage parameter Lambda = 0.5, since we simulated the same multimodal signals for ATAC and RNA and we expect to detect them in both omics. We reported these details also in the main text (lines 119-121). We also added for each case study the information regarding the lambda and model selection procedure used to run CONGAS+. In detail, while for the gastric and prostate cell lines we employed lambda = 0.5, for the B-cell lymphoma we exploited lambda = 0.2. To select these values we followed the procedure that we added in Section 1.3 of the Supplementary Information. We refer to this procedure also in the response to the next point (point n. 3)

3) The impact of \lambda is analysed but no clear conclusion is given. For a given real dataset, how should the user choose \lambda?

We thank the reviewer for pointing out this issue in the explanation. We acknowledge the fact that RNA distributions may be affected by multiple confounding factors, and thus we added the lambda parameter to fine tune the inference in settings where copy number events are evident only in one of the two modalities. In the Supplementary Information we added Section 1.3 “Tuning the hyperparameter lambda”, that provides guidelines to perform the tuning.

4) The results are not convincing, because for both of the two real datasets the model failed to find any CAN sub-clones. The analysis of BCC data focuses on showing that the model manages to separate tumor from cancer cells (finding 2 clusters). Therefore, the summary sentence “Overall, this shows that with a joint inference on the ATAC and RNA modalities we can detect the clonal structure of the dataset " is an overstatement. For the second dataset, the model finds a single cluster for the tumor cells (no sub-clones) and two clusters for normal cells are identified. Both the first and the second part are very suprising. The authors spend some discussion explaining why there could be normal clusters, but this is pure speculation, without any additional data for validation. At the same time, the single cluster for tumor cells is even more suprising, and this is a fact that is not explained. Maybe these two datasets are some exception to the rule, but the model should inste
ad be applied to something more typical, where more clones could be found. Otherwise its functionality beyond separating tumor from normal cells is not shown. Definitely, single cell DNA seq datasets suited for CAN calling, such as DLP, show much more clones and a large CAN variety among cells (see https://www.nature.com/articles/s41586-022-05249-0, Fig 1e).

We added in the main text an example of a dataset of a Gastric Cell line SNU601 which has been profiled with scDNA sequencing, scRNA and scATAC sequencing in [1] and [2], and it is thus a dataset that can be used to validate the ability of CONGAS+ to correctly infer the clonal structure in the data. The authors in [2] define copy number subclones using CNAs derived from the analysis of scDNA data, and then map each single-cell ATAC profile to the corresponding copy number subclone.

We have exploited the same data to test the ability of CONGAS+ in identifying multiple tumor sub-clones: we used the copy number profile of the most abundant population to define segment breakpoints and to set the prior on the copy number inference, and we show that we are able to detect 4 out of 5 subclones both in scRNA and scATAC. We think that this is good because we are well aware that detecting CNAs from RNA/ATAC is much more difficult than from low-pass DNA sequencing. We also show that we are able to associate each inferred cluster to a ground truth clone by looking at the cluster composition in terms of ground truth labels, and by comparing the distance between inferred and ground truth copy number profiles.

In both cases we obtained the same correspondences, meaning that CONGAS+ was effective in grouping cells together and inferring the correct copy number profiles of the multiple clones. For a detailed explanation please refer to the results section in the main text (lines 186-228).

With respect to the concerns raised on the multiome B-cell lymphoma data, please refer to the second point in the response to reviewer 2.

[1] Wu CY, Lau BT, Kim HS, Sathe A, Grimes SM, Ji HP, Zhang NR. Integrative single-cell analysis of allele-specific copy number alterations and chromatin accessibility in cancer. Nat Biotechnol. 2021 Oct;39(10):1259-1269. doi: 10.1038/s41587-021-00911-w. Epub 2021 May 20. PMID: 34017141; PMCID: PMC9127226.
[2] Andor N, Lau BT, Catalanotti C, Sathe A, Kubit M, Chen J, Blaj C, Cherry A, Bangs CD, Grimes SM, Suarez CJ, Ji HP. Joint single cell DNA-seq and RNA-seq of gastric cancer cell lines reveals rules of in vitro evolution. NAR Genom Bioinform. 2020 Jun;2(2):lqaa016. doi: 10.1093/nargab/lqaa016. Epub 2020 Mar 14. PMID: 32215369; PMCID: PMC7079336.

5) Maybe the problem with finding clones comes from the fact that CONGAS+, as mentioned briefly, “By default, scans for large CNAs at the resolution of chromosome arms”. Why is this so? This is not explained and not clear from the text. In fact, DNA-seq data shows that much smaller events are prevalent and important and form sub-clones.

In order to show the resolution of CONGAS+ solution in the absence of a WGS assay, we added in the supplementary material an example of the dataset from a gastric cell line with matched scATAC, scRNA and scDNA from [1] and [2]. Instead of exploiting the whole genome experiment, we ran CONGAS+ using chromosome arms as segment coordinates, and compared our result with ground truth labels.

We found that CONGAS+ is able to exploit the signal present on a subset of chromosome arms to separate the major clusters present in the data. In the future, we plan to develop a segmenter to lift this limitation (mentioned in the Discussion, lines 344-349), but so far we feel that this improvement is beyond the scope of this work. For more detailed information regarding this case study we refer to the supplementary information.

We finally note that in the absence of segmentation from bulk, as we do in some examples, we use chromosome arms (or cytogenetic bands) as default segments, with the goal of identifying large copy numbers. While this is not always ideal, the choice of the segmentation is left to the users, who can set a lower resolution (e.g., half of a chromosome arm) to run the inference and assess the presence of copy number clones in their data.

6) The introduction praises the efficiency of implementation. Unfortunately, run times are not presented. Since it is an advantage of the model, it would be great to see how short the run time is and how it scales with the cell count, number of clones, as well as the number of segments.

We have now reported in Figure 4.C the analysis of run times to show the efficiency of CONGAS+, for both cases CPU and GPU.

Minor

1) When explaining parameter inference using SVI, a set of parameters \gamma is introduced. If they are used for estimating model parameters, it should be made clear that the parameters introduced above are contained in \gamma.

We have now specified, right after introducing it (Section 1.1.4 in the Supplementary Information), that the \gamma is just a placeholder for all the variational parameters estimated during inference.
